# When Immunity Kills: The Lessons of SARS-CoV-2 Outbreak

**DOI:** 10.3389/fimmu.2021.692598

**Published:** 2021-09-24

**Authors:** Yassine Taoufik, Marie-Ghislaine de Goër de Herve, Stéphanie Corgnac, Antoine Durrbach, Fathia Mami-Chouaib

**Affiliations:** ^1^ Institut National de la Santé et de la Recherche Médicale (INSERM) Unité Mixte de Recherche (UMR) 1186, Integrative Tumor Immunology and Immunotherapy, Gustave Roussy, Fac. de Médecine - Univ. Paris-Sud, Université Paris-Saclay, Villejuif, France; ^2^ Department of Hematology and Immunology, Assistance Publique – Hôpitaux de Paris, Université Paris-Saclay, le Kremlin-Bicêtre, France; ^3^ Department of Nephrology, Assistance Publique – Hôpitaux de Paris, Hôpitaux Universitaires Henri Mondor, Créteil, France

**Keywords:** SARS-CoV-2, immunopathology, T cell response, complement activation, B cell response

## Abstract

Since its emergence at the end of 2019, SARS-CoV-2 has spread worldwide at a very rapid pace. While most infected individuals have an asymptomatic or mild disease, a minority, mainly the elderly, develop a severe disease that may lead to a fatal acute respiratory distress syndrome (ARDS). ARDS results from a highly inflammatory immunopathology process that includes systemic manifestations and massive alveolar damages that impair gas exchange. The present review summarizes our current knowledge in the rapidly evolving field of SARS-CoV-2 immunopathology, emphasizing the role of specific T cell responses. Indeed, accumulating evidence suggest that while T-cell response directed against SARS-CoV-2 likely plays a crucial role in virus clearance, it may also participate in the immunopathology process that leads to ARDS.

## 1 Introduction

Coronaviruses are a family of single-strand positive RNA enveloped viruses that infect a wide range of hosts. To date, seven viruses are known to infect humans. They include four common human coronaviruses: 229E and NL63 (alpha coronavirus) and OC43 and HKU1 (beta coronavirus). Those endemic viruses cause, in most cases, mild to moderate upper-respiratory tract illnesses (common cold). Human coronaviruses also include three highly pathogenic epidemic beta coronaviruses:

1. Severe Acute Respiratory Syndrome Coronavirus (SARS-CoV) (1), the epidemy of which emerged in China in 2002 and ended in 2003.
2. Middle East Respiratory Syndrome Coronavirus (MERS-CoV), that appeared in the Middle East in 2012 (2).
3. The highly contagious SARS-CoV-2, which outbreak started in the province of Wuhan in China at the end of 2019, and which spread worldwide at a very rapid pace.

**Figure 1:**
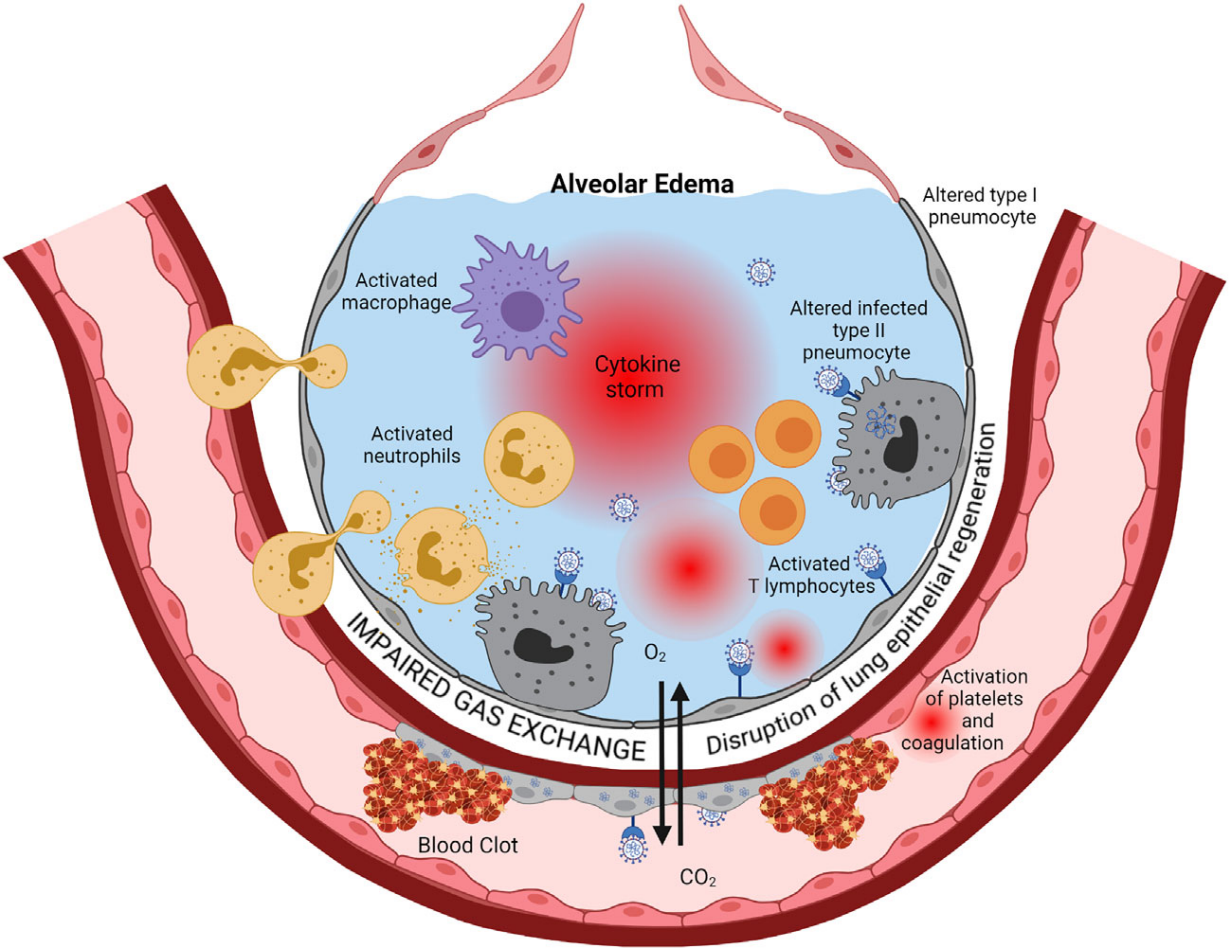
Immunopathology of ARDS. Cytopathic SARS-CoV-2 infection of pulmonary epithelial cells and endothelial cells is the starting point of a series of cascading pro-inflammatory events leading to ARDS eventually. Viral replication, *via* activation of the innate immune response components such as TLR, triggers the production of interferons, inflammatory cytokines, and chemokines. This inflammation induces infiltration into the alveoli and activation of neutrophils, macrophages, and lymphocytes. Activated immune cells produce large amounts of pro-inflammatory cytokines such as IL-6, IL-1β, TNF-α, and IL-8 that exacerbate local cell recruitment and activation, leading to a “cytokine storm” through this amplification loop. The increase of capillary permeability causes pulmonary edema. Thrombosis occurs *via* several mechanisms, including injury of endothelial cells by the virus and subsequent activation of the coagulation cascade, activation of neutrophils that secrete procoagulant factors, and activation of complement that leads to activation of platelet and coagulation. Regeneration of the lung epithelia is impaired. This cascade of events ends in a severe impairment of Gas exchanges between alveoli and lung capillaries, causing hypoxemia. Created with BioRender.com.

Those three highly pathogenic coronaviruses predominantly infect the lower respiratory tract, mainly alveolar epithelial cells. They may cause fatal pneumonia associated with increased production of pro-inflammatory cytokines and chemokines, lung infiltration by mononuclear inflammatory cells, acute lung injury with massive diffuse alveolar damage, leading to an acute respiratory distress syndrome (ARDS) (1, 2) (see **Figure 1**).

Infection with the new coronavirus SARS-CoV-2 can be divided into three stages: 1) an asymptomatic incubation period with or without detectable virus, 2) a non-severe symptomatic period with a detectable virus, and 3) a severe symptomatic period with the predominance of respiratory symptoms and variable viral loads in nasopharyngeal samples (3, 4). In some patients worsening may occur despite a decrease in viral load in nasopharyngeal samples (5), while in others, it is associated with high viral loads (3–5). Not all patients progress to the severe phase of the disease. The proportion of patients from the general population, regardless of age, who advance to the critical stage, ranges from 8 to 15% (6, 7). However, there is a higher risk of progression for patients over 65 years old (6). There is also a high prevalence of obesity and hypertension in SARS-CoV-2-infected patients who require invasive mechanical ventilation (8). The risk of progression to severe respiratory complications and death is also higher in patients with cancer (7, 9), including hematological malignancies (10).

While clinical worsening with severe respiratory complications is related to exacerbated immunopathology, it is not necessarily associated with high viral loads. Virus replication in alveolar epithelial cells may trigger in some patients an uncontrolled immunopathology process that continues to exacerbate while viral burden reduces. In SARS-CoV-2 infected patients, ARDS occurs approximately between day 9 and day 12 following the onset of symptoms (11). It is associated with biological hallmarks of intense inflammation (e.g., increase of serum ferritin and CRP), coagulation activation (e.g., increase of d-dimers), and heart damage (e.g., increase of Troponin). The incidence of thrombotic complications appears particularly high in intensive care unit patients with SARS-CoV-2, with pulmonary embolism being the most frequent, ranging from 20.6 to 31% (12, 13).

## 2 Immunopathology of Acute SARS-CoV-2 Infection

### 2.1 Interactions Between SARS-CoV-2 Infection and ACE2

The first contact of SARS-CoV-2 with its human target cell is through the interaction of the spike protein (S Protein), a primary site of neutralization (14), and the SARS-CoV receptor angiotensin-converting enzyme (ACE) 2 (14, 15). ACE2 is also the entry receptor of SARS-CoV but not that of MERS-CoV, the functional entry receptor of which is DPP4 (dipeptylpeptidase 4), also known as CD26 (16).

Biophysical and structural evidence suggest that SARS-CoV-2 S protein binds ACE2 with higher affinity than SARS-CoV S protein (17). Several SARS-CoV-2 major variants with mutations within the amino-terminal domain (NTD) and the receptor-binding domain (RBD) of the S protein that increase affinity for ACE 2 have emerged since the beginning of the outbreak (18). For both SARS-CoV and SARS-CoV-2, the S protein is cleaved by the host cell serine protease TMPRSS2 (14), allowing its conformational change and exposition of the fusion peptide required for virus delivery into the cell. Also, neutralizing antibodies against the SARS-CoV S protein may provide some protection against SARS-CoV-2 (14), although limited (17). ACE2 has a remarkable abundance at the cell surface of lung alveolar epithelial cells (pneumocytes type 1 and 2) and enterocytes of the small intestine, two cell types in contact with the external environment (19). It is also broadly present on arterial and venous endothelial cells and arterial smooth muscle cells in numerous organs, including lung, heart, liver, gut, kidney, and brain (14). TMPRSS2 has a wide distribution in epithelia, including lung, gut, pancreas, kidney, prostate, testis, and is regulated by androgen, which may account for a higher incidence of severe SARS-CoV-2 infection in adult males (20, 21).

ACE2 is a major negative regulator of the renin-angiotensin-aldosterone system (RAAS), a cascade of vasoactive peptides. ACE2 degrades angiotensin II (AngII) to angiotensin (1–7), reducing its action on vasoconstriction, sodium retention, and fibrosis. Previous works *in vitro* on human cells and *in vivo* on experimental mouse models have shown that SARS-CoV interaction with the S protein leads to ACE2 downregulation (22, 23). ACE2 is a critical negative regulatory factor for the severity of lung edema and acute lung failure (22, 23). Recombinant ACE2 can protect mice from severe acute lung injury (22). AngII acts as a vasoconstrictor and pro-inflammatory factor after binding to angiotensin receptor type I (AT1R), mainly on non-immune cells (24). The possible downregulation of ACE2 after interaction with the S protein of SARS-CoV-2 (25) may increase the pro-inflammatory action of AngII. The AngII-AT1R axis in the respiratory system can activate both NF-κB and STAT3 that act synergistically and trigger an interleukin-6 (IL-6) signaling amplification loop in non-immune cells known as IL-*6-mediated inflammation amplifier* or IL-6 amplifier for short (26). IL-6 amplifier leads to the production of numerous pro-inflammatory cytokines and chemokines, including IL-6, and the recruitment of immune cells that exacerbate inflammation (26). The age-dependent increase of COVID-19 mortality may be related to the age-dependent enhancement of the IL-6 amplifier (27).

### 2.2 Interferon Response and Cytokine Release During Acute SARS-CoV-2 Infection

The onset of symptoms following the incubation period to SARS-CoV-2 is associated with intense virus replication (28). At that time, viral proteins and RNA had activated the innate components of the immune response, including toll-like receptors (TLRs) and RIG-I-like receptors (RLRs), with the production of interferons (IFN), inflammatory cytokines, and chemokines. Viral RNA internalized into endosomes may activate TLRs and promote the secretion of pro-inflammatory cytokines, such as tumor necrosis factor-α and IL-6 (29, 30). IFNs are critical components of the innate immune response that install an antiviral state in uninfected cells, impair viral replication in infected cells, and activate various innate and adaptative immune cells (30). Type I IFN and inflammatory cytokines and chemokines may also have a role in the rapid and transient lymphopenia that accompanies many acute viral infections by favoring lymphocyte redistribution (31). It has been reported a suppression of type I IFN signatures in peripheral blood of SARS-CoV-2-infected patients with severe disease, along with decreased plasma IFN-α concentrations, compared to patients with mild to moderate disease (32). By contrast, severe and critical patients had enhanced pro-inflammatory IL-6 and TNF-α–responses (32). SARS-CoV-2 may inhibit IFN-α production *via* the inhibitory effects of non-structural proteins 6 and 13 (nsp6 and nsp13) and open reading frame 6 (ORF6) on IRF3, a key transcriptional factor involved in the IFN-α activation (33). Moreover, several SARS-CoV-2 proteins, including nsp6 and nsp13, inhibit IFN-α signaling by blocking (STAT1)/STAT2 phosphorylation and/or its nuclear translocation (33). Those results suggested that systemic type I IFN response may have a positive effect on SARS-CoV-2 infection. However, the effects of IFNs appear much more complex (34). Indeed, contrasting with the benefit of this systemic type I IFN response, a correlation has been shown between the high levels of type I and type III IFNs, in particular IFN-λ mRNA, in naso-oropharyngeal and bronchoalveolar lavage fluid samples and disease morbidity in SARS-CoV-2–infected patients (35). While type I IFNs are widely expressed, IFN-λ responses, in terms of cytokine production and receptor expression, appear restricted to a limited number of cell types that include epithelial cells (34). The signaling pathway of IFN-λ in epithelial leads to the induction of a group of IFN-stimulated genes (ISGs), and the tumor suppressor p53, the latter limiting viral replication by enhancing IFN signaling and inducing cell cycle arrest of infected cells (34, 36). In mice infected with influenza virus, IFN-λ may impair proliferation of lung epithelial cells during recovery (35, 37), as well as differentiation of alveolar epithelial progenitor cells to secretory and multiciliated cell subtypes (37). IFN-λ produced by dendritic cells in the lungs of mice exposed to a synthetic viral RNA may damage the lung epithelium, favoring bacterial superinfection (35). Those results suggest that while systemic type I IFN may help limit virus spreading, IFN-λ, which mainly acts on mucosal surfaces, may disrupt lung epithelial regeneration during recovery, exacerbating respiratory disease. One limitation of those results is that they were mainly obtained in mice infected with influenza virus, and this experimental model may not necessarily reflect the features of SARS-CoV-2 infection in humans.

The severe phase of SARS-CoV-2 infection is associated with high blood levels of inflammation markers, such as ferritin and CRP (38–40), and a cytokine release syndrome with the production of a panel of pro-inflammatory and anti-inflammatory cytokines, including IL-1β, TNF-α, IL-6, IL-7, IL-8, IL-9, GM-CSF, G-CSF, MCP-1, IP-10, IFN-α, and IL-10 and TGF-β (3, 40–44). Noteworthy, TGF-β may trigger a process of pulmonary fibrosis (44, 45). The increase of CRP suggests the important secretion of IL-6. Such observations raised the possibility of the therapeutic use of anti-IL-6 and anti-IL-1β monoclonal antibodies to treat the severe phase of SARS-CoV-2 infection. Tocilizumab is a humanized monoclonal antibody to the IL-6 receptor (IL-6R), used to treat rheumatoid arthritis and, more recently, severe cytokine release syndrome caused by treatment with chimeric antigen receptor T cell (CAR-T) immunotherapy. Initial studies suggested that Tocilizumab may have some efficacy in patients in the severe phase of SARS-CoV-2 (46–49). However, subsequent randomized clinical studies showed disparate results (50). While in some studies, IL-6R antagonists improved outcomes (51, 52), including survival (52), others failed to demonstrate significant clinical benefit (53, 54).

### 2.3 Complement Activation During Acute SARS-CoV-2 Infection

**Figure 2:**
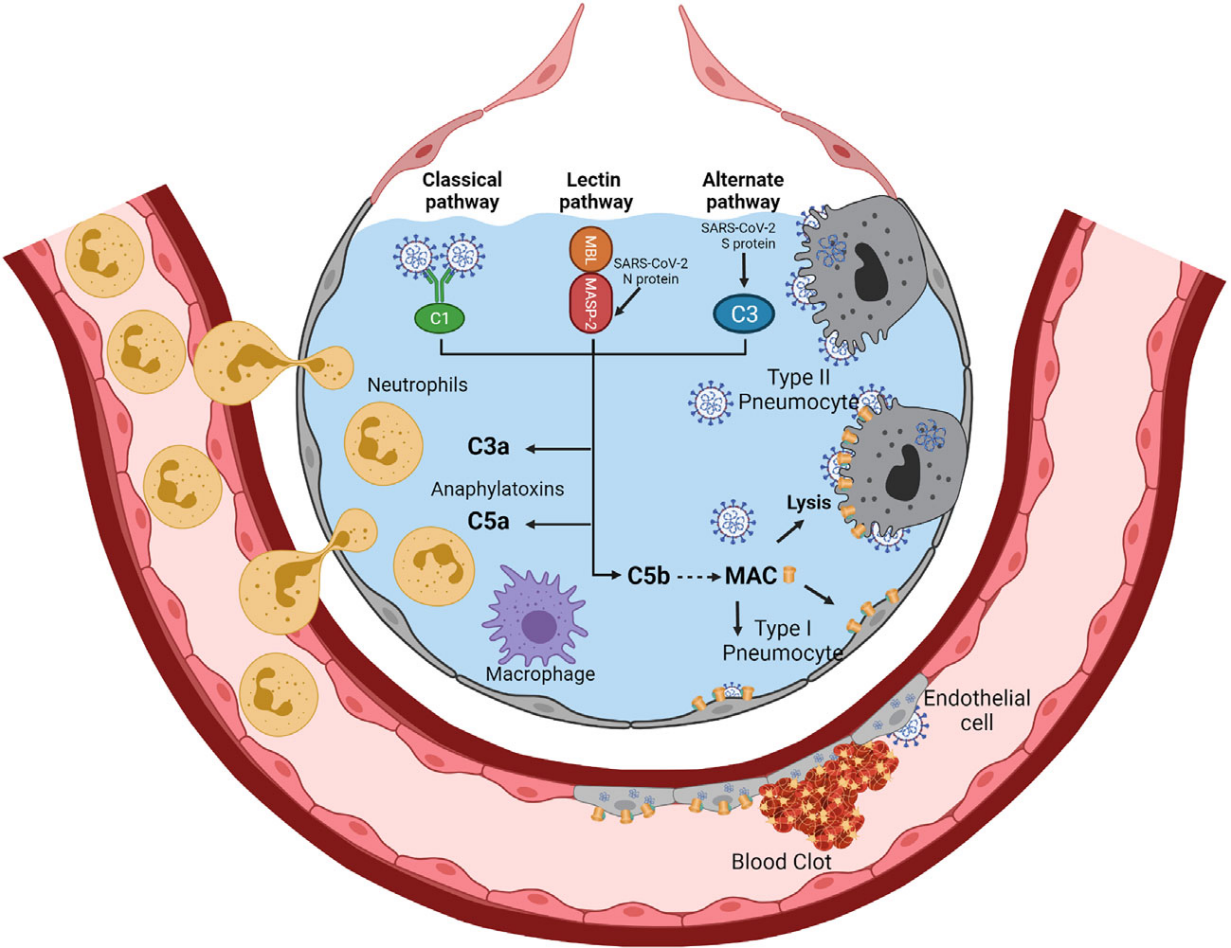
Complement activation during SARS-CoV-2-related ARDS. SARS-CoV-2 infection of alveolar epithelial cells (type II and type I) and endothelial cells may trigger complement activation *via* the three pathways of complement activation. The binding of the SARS-CoV-2 nucleocapsid protein to MASP-2 may activate the lectin pathway of complement activation. Activation of the alternative pathway could be facilitated by the lack of complement inhibitors (CD46, CD55, and CD59) on infected alveolar cells and virions. IgM to SARS-CoV-2 may efficiently activate the classical pathway. The three complement activation pathways converge to generate the C3a and C5a anaphylatoxins and the Membrane Attack Complex (C5b-C9) (MAC). C3a and C5a are potent chemoattractants and inflammatory mediators that recruit and activate neutrophils and monocytes/macrophages. Deposition of MAC on the membranes of alveolar epithelial cells and endothelial cells leads to cell lysis. Complement overactivation may promote hypercoagulability and thrombosis *via* several mechanisms, including activation and injury of endothelial cells, platelet aggregation and platelet prothrombinase activation, and inhibition of fibrinolysis. Also, MASP-2, in addition to trigger complement activation, may cleave prothrombin to form activated thrombin. Created with BioRender.com.

In four patients with severe SARS-CoV-2, requiring non-invasive mechanical ventilation, Eculizumab, a C5 blocking monoclonal therapeutic antibody, led to rapid clinical improvement (55). This suggested that blocking complement activation could be beneficial for patients infected with SARS-CoV-2. In patients with SARS-CoV-2–associated ARDS and purpuric skin rash, deposits of terminal complement components C5b-9 (membrane attack complex), C4d, and mannose-binding lectin (MBL)-associated serine protease (MASP)2 were found in both pulmonary and dermal microvessels, suggesting systemic activation of the alternative and lectin-based complement pathways (56). Higher complement activation products in blood, including soluble C5b-9, correlated with and predicted increased disease severity (57). Activation of the complement may be the consequence of the binding of the coronavirus nucleocapsid protein to the mannose-binding lectin (MBL)-associated protease-2 (MASP-2), a serine protease of the lectin pathway of complement activation (58, 59). The nucleocapsid protein appears highly conserved between SARS-CoV, MERS-CoV, and SARS-CoV-2 (60–62). Direct activation of the alternative complement pathway by the SARS-CoV-2 S protein has also been suggested (63), especially that SARS-CoV-2 infection down-regulates expression of the complement activation inhibitors CD46, CD55, and CD59 (64, 65). In an experimental mouse model of MERS-CoV, increased concentrations of the C5a and C5b-9 complement products were found in sera and lungs, respectively, and inhibition of complement activation with anti-C5a reduced lung damage (41, 64, 66)**. **In another experimental mouse model of SARS-CoV infection, C3 invalidation was associated with substantial reductions in tissue lesions and recruitment of inflammatory cells in the lungs, suggesting that the complement may play a significant role in the early step of inflammation (67). Interestingly, in this experimental model, C3 invalidation did not increase virus replication, suggesting that complement-dependent opsonization mechanisms are not critical for controlling virus replication (67). The levels of soluble C5a were increased in the blood and bronchoalveolar lavage fluid according to the severity of SARS-CoV-2 infection (68). In parallel, high expression levels of C5a receptor 1 (C5aR1) were found in blood and pulmonary myeloid cells (68). This suggested that blocking the C5a–C5aR1 axis may have a therapeutic interest in preventing excessive lung myeloid cell infiltration and inflammation (68). Another point of increasing attention is the effect of complement overactivation on thrombosis. Indeed, complement activation may favor hypercoagulability and thrombosis *via* several mechanisms that include activation and injury of endothelial cells, platelet aggregation and activation of platelet prothrombinase, as well as inhibition of fibrinolysis (69–72). Also, MASP-2, in addition to trigger complement activation, may cleave prothrombin to form activated thrombin (73). The potential mechanisms of complement activation during SARS-CoV-2 and ARDS are summarized in **Figure 2**.

### 2.4 Antibody Responses During Acute SARS-CoV-2 Infection

Initial observations suggested that the median time to detect specific IgM and IgA in serum is 5 days following symptom onset, while the median time to detect IgG is 14 days (74). IgA likely dominate the early SARS-CoV-2 antibody response in serum, saliva, and broncho-alveolar fluid (75). Within 19 days following symptoms onset, 100% of patients had detectable anti-SARS-CoV-2 IgG (76). Among antibodies to SARS-CoV-2, neutralizing antibodies (NAbs) prevent cell infection by disrupting the interaction between the S protein and ACE2. NAbs are directed against either the RBD or the NTD, both being located on the S1 subunit of the S protein (77, 78). NAbs are detectable in 85% of patients after recovery from SARS-CoV-2 infection (79, 80). This proportion was higher among patients with severe SARS-CoV-2 infection and lower in asymptomatic infections (80).

In vaccinated individuals or individuals who have recovered from SARS-CoV-2 infection, NAbs likely play an important role in the protection from symptomatic infection or re-infection, respectively (81, 82). NAbs may be produced early during acute SARS-CoV-2 infection. Neutralizing IgA, directed against the RBD, were detected in a significant proportion of patients in the first week after the onset of symptoms (75). Neutralizing IgA peaked 3 weeks after the onset of symptoms then decreased by week 4 while anti-RBD IgG reached a plateau (75). However, anti-RBD IgA may persist for longer periods in saliva (75).

In severe SARS-CoV-2 infection, neutralization potency of anti-RBD antibodies predicted survival (83). In outpatients with early SARS-CoV-2 infection (less than one week after the onset of symptoms), administration of monoclonal IgG directed against the S protein (Casirivimab/Imdevimab combination or Bamlanivimab) led to rapid decline of the viral load in the nasopharynx (84, 85). No added benefit of those therapeutic antibodies was found in sicker hospitalized patients (86). Possibly, because in later stages of the disease, inflammation and thrombotic events play a more significant role in the patient’s outcome than SARS-CoV-2 replication (86).

In some cases, antibodies to viruses may also promote inflammation and immunopathology *via* a process known as antibody-dependent enhancement (29, 87). Such pathogenic antibodies likely mainly include low affinity and non-neutralizing antibodies (29). Pathogenic antibodies may target S protein of SARS-CoV on epitopes others than neutralizing sites such as RBD (29). Immune complexes involving pathogenic antibodies may exacerbate inflammation and promote tissue injury by activating TLRs in endosomes following internalization *via* FcR in immune myeloid cells (29). Pathogenic antibodies may also include IgM that activate efficiently the complement classical pathway (29). In experimental models of SARS-CoV-infected macaques, anti-S protein IgG promoted production by alveolar macrophages of monocyte chemoattractant protein (MCP)-1 and IL-8 in productively infected lungs (88). Lung macrophages also displayed a loss of TGF-β and high expression of IL-6 (88). *In vitro*, sera from patients who died of SARS-CoV infection enhanced SARS-CoV-induced MCP-1, and IL-8 production by monocytes-derived macrophages was observed and was reduced upon FcγR blockade (88). In SARS-CoV infected macaques, the passive transfer of antibodies against the S protein prevents SARS-CoV entry in pneumocytes (88). However, passively transferred anti-spike IgG also increased macrophage infiltration and favored the occurrence of more severe pneumonia (88). Whether such pro-inflammatory pathogenic antibodies are present during acute SARS-CoV-2 infection in humans and play a significant deleterious role is a point of interest that deserves detailed investigations. Moreover, the potential pathogenic role of antibodies against human endemic « common cold » coronaviruses, that may bind SARS-CoV-2 with low affinity, remains to be determined.

During acute SARS-CoV-2 infection, in patients with severe disease, an absence of germinal centers has been shown along with a reduction of germinal center B cells, but the preservation of AID-expressing B cells (89, 90). The loss of germinal centers may be related to a failure of Bcl6+ T follicular help cell (Tfh) differentiation (90). This impairment in Tfh differentiation could be secondary to changes in the extra-follicular cytokine milieu, including high levels of TNF-α induced downstream of a strong Th1 response. Those defects in B cell differentiation in patients with severe SARS-CoV-2 infections have also been associated with increased proportions of SARS-CoV-2-reactive cytotoxic Tfh cells that may kill B cells and dampen germinal center responses (91). Loss of germinal centers may not enable the generation of long-lived memory or high-affinity SARS-CoV-2 specific B cells (90). Whether such a loss of germinal centers may also affect patients with a mild disease remains to be determined.

### 2.5 T Cell Responses During Acute SARS-CoV-2 Infection

A specific T cell response is required for virus clearance in many viral infections. The conventional paradigm is that following viral replication in a specific tissue, such as alveolar epithelial cells, local dendritic cells that have taken up viral antigens migrate to the draining lymph nodes and prime naive CD4 and CD8 T cells to generate specific primary effectors and later highly functional memory cells. Antigen-specific CD8 effector T cells rapidly expand, acquire the ability to produce cytokines, such as IFN-γ and TNF-α, and cytotoxicity-associated molecules, such as perforin and granzymes, then migrate to lungs, to exert cytotoxicity toward infected cells (92).

A central biological hallmark of severe SARS-CoV-2 infection is lymphopenia, which correlates with poor clinical outcome (93, 94). Although lymphopenia affects particularly T cells (95), B cell and NK cell counts are also decreased in patients with severe SARS-CoV-2 infection (96). Following recovery, lymphocyte counts return to normal values in most cases (95, 96). Lymphopenia is also a common observation in several acute viral respiratory infections, including SARS-CoV and MERS-CoV infections (97, 98). Lymphopenia may be related to lymphocyte redistribution that involves both retentions in lymphoid tissues and infiltration into target tissues of virus replication (31). Retention in lymphoid tissues may favor antigen priming of naive T cells by dendritic cells to initiate primary effector expansion and the process of memory generation. Lymphopenia may exacerbate, in severe forms of the infection, according to the level of lung inflammation (6). Divergent observations on lung lymphocyte infiltration during the severe phase of SARS-CoV-2 infection were reported. Some authors reported that immune cells infiltrating lungs mostly are macrophages and neutrophils along with a few lymphocytes; most of them were CD4 T cells (99). Others authors reported that lung mononuclear cell infiltrates were dominated by lymphocytes (45).

Observations on patients with severe SARS-CoV infection indicated a full restoration of CD8 lymphopenia between the third and the fifth week after disease onset. By contrast, a full restoration of CD4+ lymphopenia was slower, as CD4 cell count remains lower than in controls three months after the onset of disease, although a rapid and significant recovery was observed since the third week (31, 100). Such rapid recovery points out tissue sequestration. However, other mechanisms for lymphopenia may be involved including T depletion associated with the lymphoid organ necrosis reported during SARS-CoV-2 infection (99).

Transcriptomic analysis of bronchoalveolar lavage fluid and peripheral blood mononuclear cells in patients infected with SARS-CoV-2 suggested that this virus may induce apoptosis and P53 signaling pathway in lymphocytes (44). The capacity of SARS-CoV-2 to infect lymphocytes *in vivo* remains unclear. There is no significant expression of ACE2 on T and B cells (62). Observations of T and B cell infection by SARS-CoV have been reported (101). Other receptors may be possibly involved in the entry of SARS-CoV-2 into lymphocytes, including CD147 or CD26 (DDP4) (102–104). However, this remains under debate (105), and to date, ACE2 should be considered as the only entry receptor for SARS-CoV-2. Therefore, direct viral infection may not be a significant mechanism of the lymphopenia associated with SARS-CoV-2. Another potential mechanism for SARS-CoV-2 infection-induced lymphopenia is apoptosis related to inflammatory cytokines, such as TNF-α, that may trigger T cell apoptosis following interaction with TNFR1, the expression of which is increased on T cells in aged individuals (106, 107). T cell numbers in blood correlated negatively with the levels of IL-6, IL-10, and TNF-α (108).

#### 2.5.1 CD4 T Cell Responses

In patients with severe acute SARS-CoV-2 infection, a diminution of total regulatory T cells (Treg) in blood was observed (96, 109). A decrease of Treg in patients with severe disease was also found in the pool of SARS-CoV-2-specific CD4 T cells (91). Conversely, an increase in the proportion of pro-inflammatory CCR6+ Th17 within the total CD4 T cell pool was reported in a patient who died from severe SARS-CoV-2 infection with ARDS (45). CCR6 enables the homing of CD4 T cells to the lung and the gut** ** (110). Activated CD4 T cells reactive to S protein of SARS-CoV-2 are detectable in most patients with acute SARS-CoV-2 infection (96, 111). In patients with severe disease, the frequency of SARS-CoV-2–specific CD4 T cells is higher than in convalescent individuals (96). Most of those specific T cells are likely primary effectors or early memory cells in patients with acute infection, while in convalescent patients, SARS-CoV-2–specific CD4 T cells likely correspond to memory cells. S protein dominance was found for anti-SARS-CoV-2 CD4 T cells in patients with ARDS (112), while SARS-CoV-2 CD4 memory T cells may exhibit a different pattern of immunodominance, with codominance of M, spike, and N viral proteins (113). An intriguing point is that up to 60% of unexposed healthy donors also showed CD4 T cell reactivity to S protein of SARS-CoV-2, suggesting a possible cross-reactivity of memory CD4 T cells directed against the four endemic human coronaviruses (111, 113–115). This cross-reactivity may be potentially related to previous contacts with animal beta-coronaviruses (114). Therefore, CD4 T cell cross-reactivity between SARS-CoV-2 and endemic human coronavirus or even coronavirus from animals in contact with humans appears quite common. The role of these pre-existing cross-reactive memory T cell responses in the immunopathology of SARS-CoV-2 infection remains to be clarified.

It has been suggested that progression to the severe phase of SARS-CoV-2 infection is associated with a reduction in the polyfunctionality of total CD4 T cells (e.g., the ability for the same lymphocyte to produce TNF-α, IL-2, and IFN-γ) (116). Those results of apparent functional impairment of CD4 T cells in severe SARS-CoV-2 infection contrasts with other observations that functional anti-SARS-CoV-2 CD4 T cells are detectable in all ARDS patients (112). Those specific CD4 T cells mainly produce Th1 cytokines, although Th2 and Th17 cytokines were also detected (112). However, an extensive single-cell transcriptomic analysis of SARS-CoV-2 specific CD4 T cells in patients with severe SARS-CoV-2 acute infection showed that polyfunctional Th1 and Th17 cell subsets were under-represented in the repertoire of SARS-CoV-2-reactive CD4 T cells as compared to influenza-reactive CD4 T cells (91). The same study also showed in patients with severe infection a high frequency of cytotoxic CD4 T cells, that express perforin and granzyme B (GrB), including cytotoxic Tfh (91). Cytotoxic CD4 T cells (non-Tfh) may kill infected type II pneumocytes that can express class II MHC (117). Cytotoxic Tfh cells can kill B cells and dampen germinal center responses (91). As reported above, there is an impairment in Tfh differentiation and Tfh response during severe SARS-CoV-2 infection (90, 91). This defect may negatively impact not only the generation of memory B cell responses but also the generation of CD8 memory, as Tfh may also be required for the optimal differentiation of highly functional CD8 memory T cells (118).

#### 2.5.2 CD8 T Cell Responses

CD8 T cells from patients with severe SARS-CoV-2 infection showed higher activation, including higher IFN-γ and GrB expression, than patients with mild infection (116). Such strong activation may lead to high expression of exhaustion-associated receptors, including PD-1, TIGIT, and CTLA-4 (116). A massive PD-1 upregulation has been shown in patients with ARDS (108). One concern with the potential conclusions drawn from those studies is that T cell exhaustion is a process that affects T cells specific for a particular pathogen and not the whole blood T cell compartment regardless of the antigen specificity, as reported in the SARS-CoV-2 studies described above. Therefore, one cannot rule out that the increase in inhibitory receptors may be a global adaptation process of T cells to the cytokine storm rather than an intrinsic defect of the anti-SARS-CoV-2 primary T cell response. The observations on total T cells may not necessarily reflect the situation of anti-SARS-CoV-2-specific T cells, although a substantial part of activated CD8 T cells may be specific SARS-CoV-2 (119). Also, the expression of inhibitory receptors such as PD-1 on T cells during acute viral infections may not necessarily reflect a deleterious exhaustion process such as seen during chronic viral infections or cancers, but rather may be part of a physiological tuning process that adjusts T cell activation and functionality.

In a series of patients with ARDS, anti-SARS-CoV-2 specific CD8 T cells were detected in all patients admitted to an intensive care unit (112). Those CD8 T cells for which specificity included the S glycoprotein mainly have a memory effector or a terminally differentiated effector phenotype (112).

In SARS-CoV-infected patients, similar functionality, phenotype, and frequencies of virus-specific T cells were found between patients who experienced moderate and severe diseases, with most of the CD8 T cells producing only IFN-γ. A minority of specific CD8 T cells could also produce TNF-α and degranulate (CD107a+). Their frequency was higher in patients with the severe form of the disease (120). During the acute phase of MERS-CoV infection, higher frequencies of specific cytotoxic CD8 T cells have been shown in patients with moderate/severe diseases than in patients with mild disease (121). These CD8 T cells were directed mainly against the viral S protein (121), suggesting an association between the level of the specific early CD8 T cell response and the severity of the infection (121). Conversely, in experimental mouse models of MERS-CoV, viral clearance was not possible in T cell-deficient mice. However, it was possible in mice lacking B cells, indicating the crucial role of the CD8 T cell response for virus clearance (122). Similarly, 2 cases of patients with X-linked agammaglobulinemia who have developed and cleared a mild SARS-CoV-2 have been reported, suggesting that B cells and immunoglobulins are not essential for the control of virus replication during acute infection (123).

In various experimental models of acute respiratory virus infections, such as Respiratory Syncytial Virus (RSV), Influenza A Virus (IAV), Human Metapneumovirus (HMPV) and Pneumonia Virus of Mice (PVM) infections, expansion of specific CD8 T cells peaked in airways and lungs between day 8 and up to day 14 following mouse infection, and generally coincided with virus clearance (124). In antigen-naive individuals, mainly in children, during infection with respiratory viruses, including RSV, IAV, rhinovirus, and endemic coronaviruses, the peak of CD8 T cells in tracheal aspirates of primary effectors occurs around day 10 (124, 125). The key role of primary CD8 T cell effectors in mediating viral clearance during acute respiratory viral infections in children is well established (124). However, there is also evidence in experimental mouse models and, to a lesser extent, in children, of the immunopathological potential of CD8 T cells, following infection with respiratory viruses such as RSV that also infects alveolar epithelial cells (124, 126, 127). CD8 T cell-mediated immunopathology may involve the CD8 cytotoxic pathways such as perforin/GrB and Fas/Fas ligand (124) as well as the production of cytokines such as IFN-γ and TNF-α that may take part in a cytokine storm. Similarly, to CD4 T cells, memory CD8 T cells recognizing peptides conserved amongst coronaviruses are detectable in unexposed individuals. Those cells appear more abundant in patients with mild disease, suggesting a potential protective role (128). However, the situation is likely more complex and likewise their CD4 T cell counterparts, further studies are required to clarify the exact impact of those cross-reactive memory CD8 T cells.

## 3 Concluding Remarks

The immune response to acute lower respiratory viral infections must be tightly regulated, enabling pathogen elimination while maintaining crucial gas exchange. The highly inflammatory process leading to ARDS results from inappropriate regulation of the network of innate and adaptive components of the immune response triggered by SARS-CoV-2 replication in the pulmonary alveolus (see **Figures 1** **–** **3**). The anti-SARS-CoV-2 primary T cell response, which includes the complex and multi-faceted CD4 T cell response and the CD8 T cell component, likely plays an essential role in virus clearance. It may also participate in the immunopathology process leading to ARDS. Impaired regulation may affect not only the CD4 and CD8 primary effectors generated directly by SARS-CoV-2 infection but possibly also pre-existing memory T cells against endemic coronaviruses that may react to shared viral peptides. Human beta-coronaviruses may trigger long-lasting T cell memory. SARS-CoV-specific memory T cells have been detected more than 10 years after SARS (129). Anti-viral-memory T cells, including lung-resident memory T cells, may be highly reactive and may generate potent secondary responses. For instance, in mice, pre-existing anti-RSV memory CD8 T cells generated through a prime-boost vaccine strategy led to IFN-γ-mediated fatal immunopathology following virus challenge (130). Therefore, although coronavirus cross-reactive memory T cells could benefit many patients by helping to clear the virus, one cannot rule out that this pre-existing T cell memory may exacerbate immunopathology in some subsets of patients by providing uncontrolled CD4 helping signals to myeloid and lymphoid immune effectors, and by direct CD8 cytotoxicity. Patients at risk of ARDS include the elderly and patients with obesity. There is a loss in Treg functions during aging that may render aged individuals more susceptible to immunopathology (131). Obesity leads to accelerated immune senescence (132). It is also associated with chronic inflammation with dysfunction of several T cell subsets, including Treg and Th17 (132).

**Figure 3:**
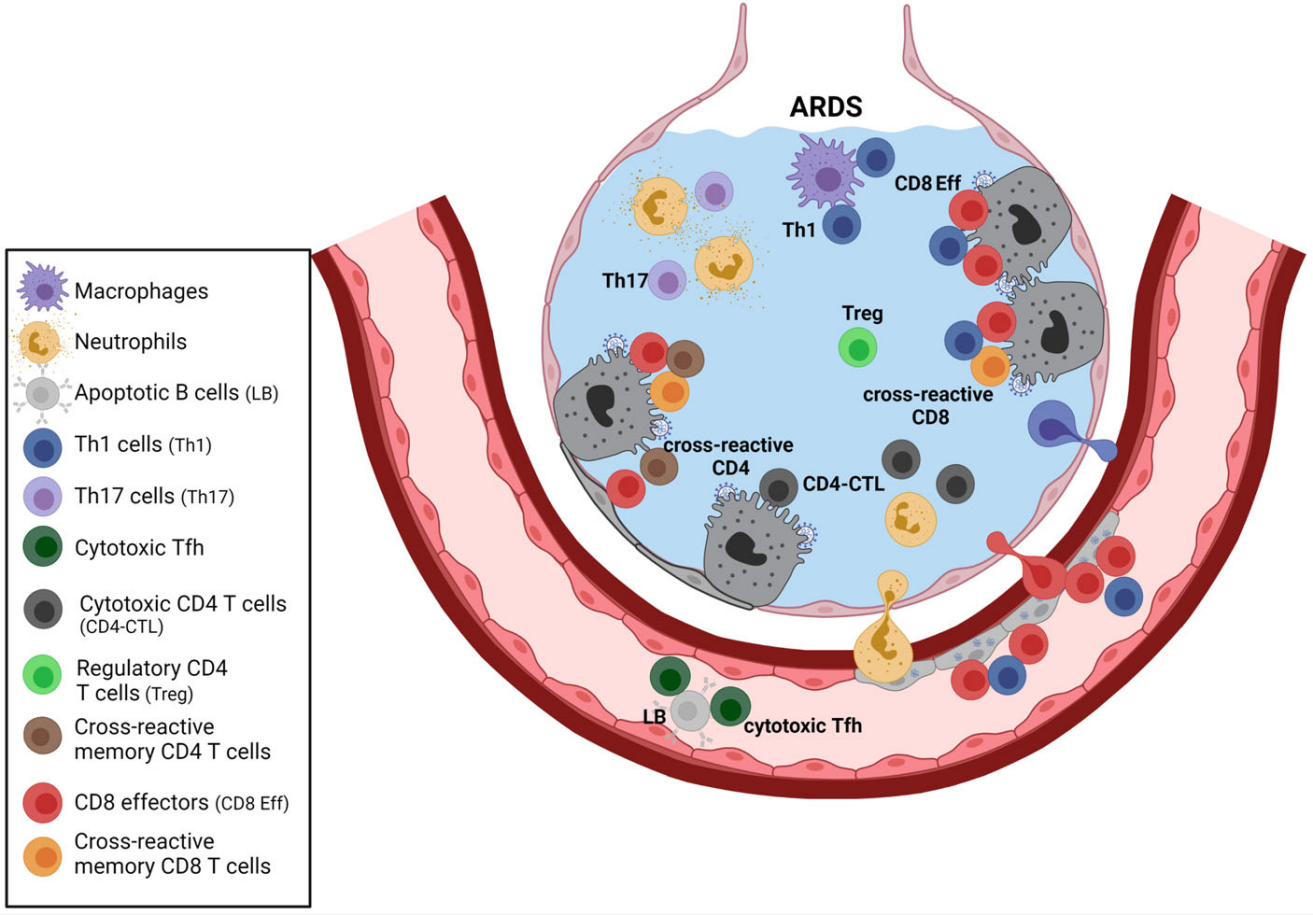
T cell responses during severe SARS-CoV-2 infection. The complex network of CD4 T cell responses during SARS-CoV-2 infection involves different CD4 subsets, including Treg, Th1 cells, Th17 cells, and some cytotoxic CD4 subsets. Th17 cells may activate neutrophils, and Th1 may provide helping signals to macrophages and CD8 effectors. Cytotoxic CD4 T cells that produce multiple chemokines (that may recruit myeloid cells) and direct cytotoxic properties have been reported in patients with severe disease. Those cytotoxic CD4 subsets could exert cytotoxicity on infected type II pneumocytes that can express MHC class II. The role of cross-reactive memory CD4 T cells generated following previous infections with endemic coronaviruses remains to be clarified. Those activated cross-reactive cells may provide help to various immune effectors, including SARS-CoV-2-specific CD8 primary effectors or coronavirus cross-reactive CD8 memory T cells (secondary effectors). Circulating cytotoxic Tfh that may kill B cells have also been described. The diminution of Treg reported in severe ARDS may exacerbate T cell mediated immunopathology. Created with BioRender.com.

A better understanding of the regulatory processes within the complex network of immune effectors acting in pulmonary alveoli (see **Figures 1** **–** **3**), including the mix of SARS-CoV-2-specific primary T cells and beta-coronavirus cross-reactive memory T cells (**Figure 3**), is required to prevent and treat ARDS in patients at risk.

## Data Availability Statement

The original contributions presented in the study are included in the article/supplementary material. Further inquiries can be directed to the corresponding author.

## Author Contributions

YT, MGGH, SC, AD, FMC participated to the writing of the paper. All authors contributed to the article and approved the submitted version.

## Conflict of Interest

The authors declare that the research was conducted in the absence of any commercial or financial relationships that could be construed as a potential conflict of interest.

## Publisher’s Note

All claims expressed in this article are solely those of the authors and do not necessarily represent those of their affiliated organizations, or those of the publisher, the editors and the reviewers. Any product that may be evaluated in this article, or claim that may be made by its manufacturer, is not guaranteed or endorsed by the publisher.
